# Urinary Calculus in a Child Two Years of Age, Measuring One Inch and Three-quarters in Circumference, Successfully Removed by Dilatation of the Urethra

**Published:** 1861-07

**Authors:** J. Taylor Bradford

**Affiliations:** Augusta, Kentucky


ART. IV.—*Urinary Calculus in a Child Two Years of Age, measuring one inch and three-quarters in circumference, successfully removed by Dilatation of the Urethra.* By J. TAYLOR BRADFORD, of Augusta, Kentucky.

ABERNETHY has somewhere said: "If our powers should prove deficient, the spirit to make an attempt deserves praise; and in great undertakings it is enough to have shown one's self willing."

It is not always an easy matter to account for incidental convictions, when they run counter to usages and established authority. It is, or should be, however, the result of matured conviction, either founded in fact or such anatomical or physiological evidences as may carry with them a reasonable justification.

Often a slight hint, a seemingly trivial occurrence or circumstance, leads the mind into new channels and to important results. That great propelling power, which now impels almost everything over land and over sea, was first recognized "escaping from the mouth of a tea-kettle." The falling of an apple determined the laws of gravitation; the cackling of a flock of geese determined a great battle; the continued and renewed efforts of a spider to attach its web to a given point infused into a defeated general new hopes, and victory took the place of repeated defeats.

Often a word or a sentence gives color and character to our destiny. It is said that from the doubtful etymology of a single word commenced the sixty years' labor of the renowned Noah Webster; and from thirty languages of Asia and Europe he has produced a work which tinges every sentence in the English language that is fashioned by beauty or strengthened by elegance of diction.

Twenty years ago—pardon the incident, for it is practical and true to life—a valued friend of mine, naturally modest and unassuming, not remarkable for any peculiar talent, but for a good head and heart, a determined, inflexible energy to carry on his part of whatever he might undertake, commenced the practice of medicine in a small town. His competitors were all old men, and good practitioners. His strong attachment for nativity, and the commanding society by which he was surrounded, notwithstanding his offers and inducements elsewhere, caused him to continue his efforts for a time. The difficulties, said he, seemed insuperable, and the long experience and fixed families of his competitors seemed to warn him, at every turn, of his sad prospects. While debating in his own mind where he would pitch his tent, he chanced to pick up the *New York Mirror*, edited by N. P. Willis, and read from the first paragraph his eyes fell upon as follows :—

"When Daniel Webster commenced the practice of law, a friend of his attempted to dissuade him from settling in a certain location, saying the profession was too much crowded there. Webster thoughtfully, but truly, replied : 'I am going above, where the profession is not so much crowded.'"

This single sentence determined his destiny. He did go above, as did the gifted Webster ; and now I have the consolation of knowing that few of his age, on either side of the water, have made more decided marks as practitioners or surgeons. But to the subject proper.

The statistics show that in the female the division of the urethra, with or without the operation of lithotrity, lithotripsy, the recto-vesical, or the lateral operations—the latter being performed by commencing the incision at the orifice of the urethra and continuing through the neck of the bladder—carries with them a heavy per cent. as subjects of incontinence of urine. The two former, or one of the two former, is most in acceptance, both in Europe and this country; but as incontinence of urine so often follows these operations, their liability to produce inflammation in the bladder, as a consequence of the crushing, together with the liability to return from remaining fragments in the bladder, an instance of which is given by Mr. H. C. Johnson, (*London Lancet*, vol. i., 1851, p. 355,) where the disease returned two years after the operation, it is not surprising that these facts strike heavily upon our surgical acumen, and carry us back again to the base of the beginning in all surgery, the anatomical character of the part. What is it? and how does it differ from that of the male?

The numerous instances on record of the expansion and dilatability of the gall-duct, where calculi ten times the size of the natural duct are passed; the enormous expansion of the bowel by fecal obstruction, where, according to Gross's Pathological Anatomy, the "colon, duodenum, and ileum measured thirteen inches in circumference, and the quantity of fecal matter amounted to seven gallons," and the enormous dilatation of the vagina during parturition are well known.

The urethra in the female is not so "muscular" as in the male, but shorter and more dilatable; and while in the male, says Mr. Gray, in his late anatomical work, it is surrounded by a dense resisting structure, that of the female admits of "much dilatation." Influenced by these facts, briefly stated, is my only apology for the course pursued in the present case.

The patient was the daughter of Mr. Mitchell, near Foster's Landing, Bracken County, Kentucky. She was brought to Augusta, and on my first visit to her, I found her in one of those paroxysms peculiar to that class of sufferers when attempting to void urine, when stone exists. The body was thrown forward, with the hand pressed convulsively upon the region of the bladder or genital organs. For a moment she would drop upon one or both knees, then upon the nates, up again, and rotate her body laterally and forward, as though in a temporary delirium. From the severity of her suffering, and the constant straining during and for some time after micturition, extensive prolapsus of the bowel existed, as well as excoriation of the genital organs. Her father told me she had been under medical treatment for a long time by different physicians. Lately she had been treated for the "piles," under the supposition that that was the sole cause of her complaint. I told him it was stone, placed her under the influence of chloroform, introduced the sound, and was but a moment in detecting the concretion. Next day, I placed my little patient again under the influence of chloroform, and introduced into the urethra the smallest-sized bougie. Every second day thereafter, I introduced a bougie, increasing the size each time, until the largest size I could procure had been gone through with, always using the chloroform. Upon the withdrawal of the largest-sized bougie, I could easily insert the little finger into the urethra. With the assistance of Dr. Robinson and an old lady, I introduced Cooper's forceps, having a slide upon the outer surface, which allows it to be passed with the blades closed, and which, when drawn back, opens the forceps. In the centre of the instrument is a sound to test the grasping of the stone. After twenty minutes' manipulating, the stone was seized. The slide was firmly shoved down, closing the blades of the forceps over the stone, the sound used to test its security, and an effort made at extraction. The slight, if any, progress made at the first effort, induced me to apprehend I had embraced the wall of the bladder with one or both blades of the forceps. This apprehension I was rejoiced to find to be groundless, by turning the forceps over and over. A gentle but steady traction was then kept up, stopping every few moments and waiting the gradual expansion of the urethra to the desired point. This process being kept up for half an hour, the stone came in view. It seemed wonderful that it had passed any part of the canal, or could advance any more. A little further cessation, and slightly undulating traction, however, brought it up to the external margin of the urethra, but the rigidity there seemed so unyielding that I made a small niche with the point of the scissors, and out it came. The stone measured one inch and three-quarters in circumference.

The next day my little patient had complete control of the bladder, passing her urine at will. She regained her health rapidly, and now, two years since the operation, is perfectly well.

Notwithstanding my unshaken confidence in the power of the urethra, or any organ which opens externally, to yield to remarkable dilatation, yet I confess, if I had known the exact size of the stone, I would have been deterred from attempting its removal, without dividing a portion of the urethra. Now I believe, in the adult female, with care and caution, a stone three inches in circumference might be removed without the use of the knife. Mr. Furguson has extracted a stone of this size, but only by dividing the anterior half of the urethra.

The passive effect obtained over the urethra by the use of chloroform, not only in the preparatory dilatation, but at the time of the operation, is of primary importance, and, if not relaxing in itself, which, from my experience in surgery, is possible, if not probable, it exerts an influence over the entire system, which renders muscular resistance nugatory, and essential.

